# Exothermic laws applicable to the degradation of *o*-phenylenediamine in wastewater *via* a Fe^3+^/H_2_O_2_ homogeneous quasi-Fenton system

**DOI:** 10.1039/c9ra04709c

**Published:** 2019-08-21

**Authors:** Yuntao Yan, Yanpeng Mao, Yong Dong, Ke Zhang, Xiaofei Sun, Chunyuan Ma

**Affiliations:** School of Energy and Power Engineering, Shandong University 17923 Jingshi Road 250061 Jinan 250100 PR China maoyanpeng@sdu.edu.cn dongy@sdu.edu.cn +86 531 88395877 +86 531 88399372

## Abstract

We studied the exothermic laws of Fe^3+^/H_2_O_2_ homogeneous quasi-Fenton degradation of *o*-phenylenediamine in waste water, and analyzed the effects of [H_2_O_2_] and [Fe^3+^], initial reaction temperature, and other factors on the solution temperature elevation (Δ*t*), temperature elevation duration (*T*), and chemical oxygen demand degradation rate (*η*) during the degradation of the target pollutant. Our study found that [H_2_O_2_] is a major factor affecting Δ*t*, while [Fe^3+^] and *t*_0_ are the main factors influencing the exothermic reaction rate. For the conditions wherein [H_2_O_2_] is 0.2 mol L^−1^, [Fe^3+^] is 10 mmol L^−1^, pH = 7.8, initial reaction temperature is 30 °C, and reaction duration is 30 min, Δ*t* of 200 mL of 0.04 mol L^−1^*o*-phenylenediamine is 7.2 °C and *η* is 93.45%. The exothermic reaction between the free radicals (·OH and 
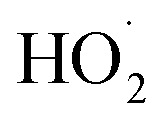
) and *o*-phenylenediamine and the exothermic reaction due to auto-consumption of free radicals are the main reasons for the increased temperature of the solution.

## Abstract 2

The external consumption and the self-consumption of reactive oxygen species are found to be the main exothermic reactions.

## Graphical abstract

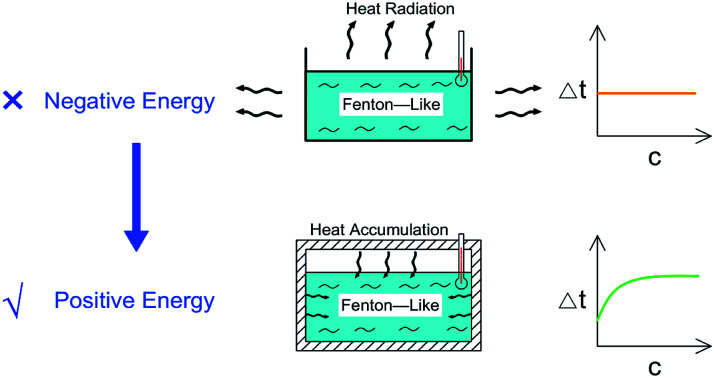

To reduce the environmental damage caused by fuel combustion, comprehensive energy utilization technology represented by heat pump has gained increasing attention. Low-input, high-grade electrical energy can be used utilized, *via* heat pumping technology, to upgrade low-grade thermal energy from urban wastewater to high-grade thermal energy that can be used during winter. The unique thermal energy collection method of this technology can effectively avoid generating harmful substances such as sulfur dioxide, nitrous oxide, heavy metals, and dust that occur during the combustion of fossil fuels;^1–3^ as such, it is a green heat supply technology that should be promoted. To ensure that the overall wastewater treatment process is not affected, existing wastewater heat pumps usually control the wastewater temperature to within 5 °C during thermal energy extraction. Therefore, studying treatment techniques in which increases in water temperature approaches or exceeds 5 °C during wastewater degradation has important significance for increasing the thermal energy output of the wastewater heat pump and utilizing the energy efficiently during wastewater treatment.

Aniline chemicals such as *o*-phenylenediamine are raw materials for the manufacture of chemicals such as dyes, pesticides, rubber, and paints, and when the sewage is discharged into the environment, it may cause potential harm to aquatic organisms and human health. It is difficult to effectively remove it by water treatment technology using traditional physical and chemical means or biodegradation. In recent years, advanced oxidation water treatment technology, which is mainly composed of strong oxidizing free radicals, has been rapidly developed. During the Fenton advanced oxidation process, the ·OH ions produced by Fenton's reagent have an extremely strong oxidation ability, which is only weaker than F_2_ among known oxidation reagents and can effectively degrade organic matter in wastewater. In addition, the entire process is simple and is often used to treat wastewater containing undegradable organic matter.^4–6^ However, conventional homogeneous Fenton systems require pH levels to be controlled below 3; thus, high volumes of acid are required for the acidification as a part of the wastewater treatment. Therefore, after degradation is complete, correspondingly high quantities of alkalis are required to neutralize the treated wastewater, which greatly increases the cost of the process.^7–9^

At present, research on Fenton technology mainly focuses on overcoming its own shortcomings such as narrow pH range.^10–12^ Deng *et al.*^13^ employed liquid-phase precipitation to prepare an iron vanadate catalyst and expanded the pH range for the degradation to 3.0–8.0. Sun *et al.*^14^ prepared an iron vanadate Fenton catalyst under neutral conditions using hydrothermal methods and tested it with relatively few samples. This resulted in better dispersion of nanorods and a higher ability of oxidative degradation at a pH range of 1.0–9.0. These heterogeneous Fenton systems employed a solid catalyst to promote H_2_O_2_ degradation to produce ·OH while maintaining the strong oxidation capability of a homogeneous Fenton system, expanding the pH range suitable for the reaction, and decreasing wastewater treatment costs. However, studies on the heat generation law of Fenton's reagents for degrading organic wastewater have not been reported.

In recent years, there has been a high volume of research and applications using Fenton or quasi-Fenton advanced oxidation technology to treat hard-to-degrade organic wastewater that primarily produces ·OH. There are several thousands of studies on this, but there is still no report of work on the exothermic law of the Fe^3+^/H_2_O_2_ homogeneous Fenton systems during wastewater treatment. To realize the comprehensive utilization of energy treatment of wastewater treatment, H_2_O_2_ and Fe^3+^ were added at various concentrations to wastewater with *o*-phenylenediamine being the target pollutant. *o*-Phenylenediamine concentration, influence of initial reaction temperature on the increase in solution temperature, duration for which the temperature remained elevated, and chemical oxygen demand (COD) degradation rate were determined. We also summarized the exothermic law of the Fe^3+^/H_2_O_2_ quasi-Fenton system during degradation of wastewater containing *o*-phenylenediamine and carried out a preliminary investigation of exothermic mechanisms.

## 1. Experiments

### 1.1 Reagents and equipment

*o*-Phenylenediamine (C_6_H_8_N_2_); 30% H_2_O_2_, Fe_2_(SO_4_)_3_, H_2_SO_4_, Ag_2_SO_4_, K_2_Cr_2_O_7_, (NH_4_)_2_Fe(SO_4_)_2_·6H_2_O, and NaOH were purchased from Sinopharm Chemical Reagent Co., Ltd.

This study used a digital display thermostatic water bath (HH-S, Tianjin Saidelisi Experimental Analyzer Factory), a pH meter (PHS-3C, Yoke Instrument Co., Ltd), a temperature and humidity recorder (TH22R-XX, Hua Han Wei Co., Ltd), a COD analyzer (JHR-2, Genstar Electronic Technology Co., Ltd), and a laboratory stirrer (JJ-1, Changzhou Boyuan Instrument Plant).

### 1.2 Experimental setup

**Fig. 1:**
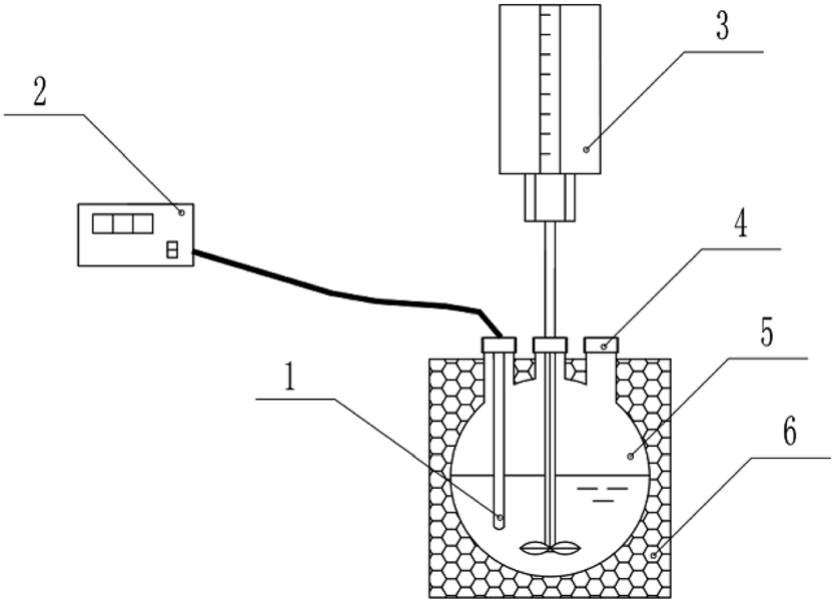
The experimental setup employed in this study. (1) Thermometer, (2) temperature and humidity recorder, (3) stirrer, (4) addition port, (5) 500 mL round-bottom flask, (6) insulation layer.

Fig. 1 shows the experimental setup. The reactions were carried out in a 500 mL round-bottom flask containing a stirring rod and a thermometer. The stirring rod was connected to a stirrer through the mouth of the flask, and the thermometer was connected to the temperature and humidity recorder with wires. The outer wall of the round-bottom flask was completely covered with a thermal insulation layer.

### 1.3 Experiment process

A certain amount of *o*-phenylenediamine was weighed, and a certain concentration of *o*-phenylenediamine simulated wastewater was prepared in a volumetric flask with deionized water. Potassium dichromate method was used to determine the COD of the solution. The pH of the simulated wastewater was adjusted using sulfuric acid and sodium hydroxide. Before the start of the degradation reaction, the volumetric flask containing the *o*-phenylenediamine solution was placed in a constant temperature water bath, and the solution was warmed to above 5 °C above the initial set reaction temperature. During the experiment, a measuring cylinder was used to add 200 mL of heated *o*-phenylenediamine solution to the round-bottom flask (Fig. 1), after which the stirrer was turned on (600 rpm). After being cooled naturally to the initial reaction temperature, H_2_O_2_ solution was added to the *o*-phenylenediamine solution first; Fe_2_(SO_4_)_3_ solid powder was added to *o*-phenylenediamine solution 2 min later while the temperature and humidity recorders were started simultaneously. This was taken to be the start of the reaction. The reaction temperature and reaction duration were recorded once every minute. During the reaction, the increase in solution temperature Δ*t* was calculated using eqn (1), while the duration of the increase in solution temperature *T* was calculated from the reaction start point to the point at which the temperature began to drop.

1 Δ*t* = *t* − *t*_0_

After the reaction began, samples were collected at 10 min intervals and a 102 moderate speed qualitative filter paper was used to filter the sample. The COD of the filtrate was measured using potassium dichromate. The level of COD reduction in the solution, Δ*Q*, was calculated using eqn (2) below:

2 Δ*Q* = *Q*_0_ − *Q*

The COD degradation rate of the solution, *η*, was calculated using eqn (3) as follows:

3 *η* = Δ*Q*/*Q*_0_ × 100%

where Δ*t* is the temperature difference before and after *o*-phenylenediamine degradation, *t*_0_ and *t* are the temperatures of the *o*-phenylenediamine solution before and after the reaction, respectively, *Q*_0_ is the initial COD of the *o*-phenylenediamine solution, *Q* is the COD value after *o*-phenylenediamine degradation, and *η* is the COD degradation rate of the *o*-phenylenediamine solution.

## 2. Results

### 2.1 Effects of the concentration of hydrogen peroxide added

**Fig. 2:**
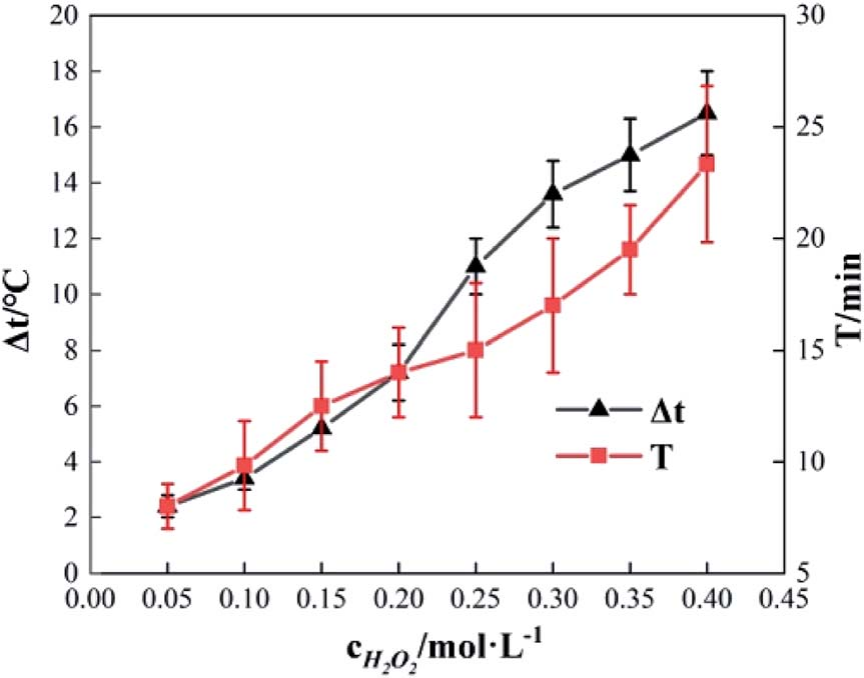
Effects of [H_2_O_2_] on Δ*t* and *T*.

**Fig. 3:**
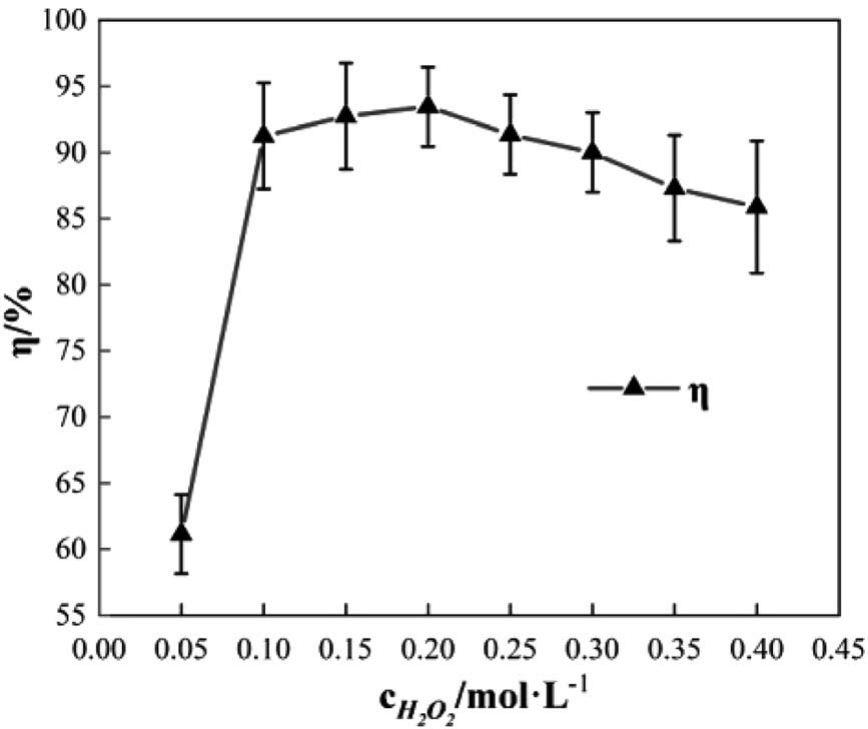
Effects of [H_2_O_2_] on *η*.

To study the effect of the concentration of H_2_O_2_ on Δ*t*, *T* and *η*, the reaction conditions were as follows: the concentration of *o*-phenylenediamine solution was 0.04 mol L^−1^, volume was 200 mL, initial reaction temperature was 30 °C, pH = 7.8, concentration of Fe^3+^ was 10 mmol L^−1^, and reaction time was 30 min. Fig. 2 and 3 show the Δ*t*/*T* curve and the *η* curve of the *o*-phenylenediamine solution with the concentration of H_2_O_2_ added. Fig. 2 shows that the temperature of the solution in the Fe^3+^/H_2_O_2_ quasi-Fenton system increased as *o*-phenylenediamine degraded. When the concentration of the H_2_O_2_ added increased from 0.05 mol L^−1^ to 0.4 mol L^−1^, Δ*t* increased from 2.4 °C to 19.6 °C and *T* increased from 8.0 min to 23.3 min. This shows that the Fe^3+^/H_2_O_2_ quasi-Fenton system exhibits a significant exothermic reaction during *o*-phenylenediamine degradation, and that the amount of thermal energy and the duration of the exothermic reaction increases as the concentration of the H_2_O_2_ added increases.

According to the Fenton mechanism,^15^ reactions depicted by eqn (4)–(9) occur simultaneously in the Fenton system. Fig. 3 shows that when the concentration of the H_2_O_2_ added increased from 0.05 mol L^−1^ to 0.2 mol L^−1^, the *η* of *o*-phenylenediamine increased from 61.13% to 93.45%. The value of *η* first increased, before decreasing to 85.89%, as the concentration of H_2_O_2_ added increased to 0.4 mol L^−1^.^16,17^ This occurred because when the concentration of H_2_O_2_ added was relatively low, almost all the ·OH and 
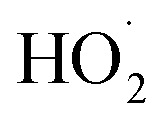
 produced during the reactions in eqn (4) and (5) also reacted with *o*-phenylenediamine as shown in eqn (6).^18^ At this point, the degradation rate increased as the concentration of H_2_O_2_ added increased. When the concentration of H_2_O_2_ added reached a certain value, ·OH and 
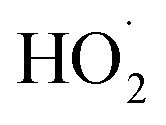
 free radicals underwent auto-consumption as per eqn (7)–(9). This resulted in ineffective decomposition of some of the H_2_O_2_,^19,20^ and consequent decrease in the degradation rate.4
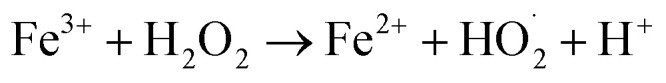

5 Fe^2+^ + H_2_O_2_ → Fe^3+^ + ·OH + HO^−^

6 RH + ·OH → H_2_O + R˙ → CO_2_ + H_2_O

7
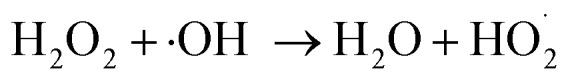
8
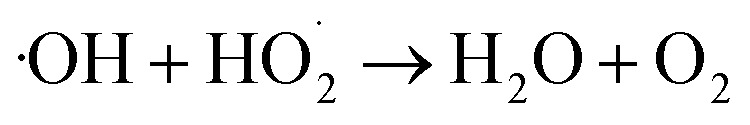
9
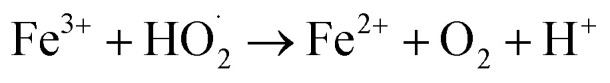


### 2.2 Effects of the concentration of Fe^3+^ added

**Fig. 4:**
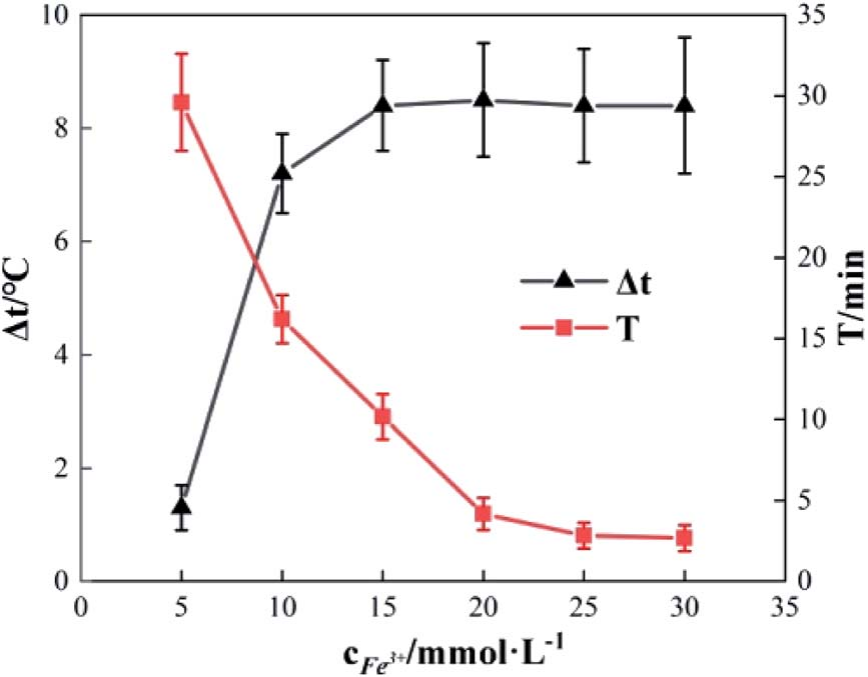
Effects of [Fe^3+^] on Δ*t* and *T*.

**Fig. 5:**
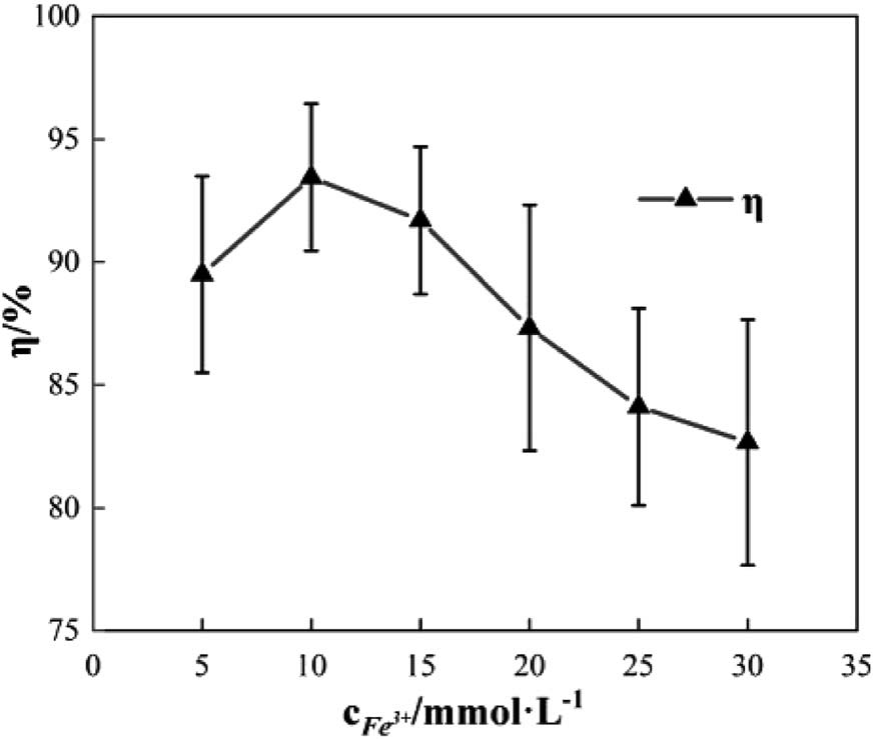
Effects of [Fe^3+^] on *η*.

To study the effect of the concentration of Fe^3+^ on Δ*t*, *T* and *η*, the reaction conditions were as follows: the concentration of *o*-phenylenediamine solution was 0.04 mol L^−1^, volume was 200 mL, initial reaction temperature was 30 °C, pH was 7.8, concentration of H_2_O_2_ was 0.2 mol L^−1^, and the reaction time was 30 min. Fig. 4 and 5 show the Δ*t*/*T* curves and *η* curves of the *o*-phenylenediamine solution with the concentration of Fe^3+^ added, respectively. When the [Fe^3+^] was 5, 10, and 15 mmol L^−1^, the corresponding Δ*t* values were 2.3 °C, 7.2 °C, and 8.4 °C, respectively. At this stage, Δ*t* increased with increasing quantity of ferric ions in the solution. Subsequently, an additional increase in the concentration of ferric ions resulted in almost no change in Δ*t*. When the [Fe^3+^] was 5, 10, 15, 20, 25, and 30 mmol L^−1^, the corresponding *T* values were 16.83, 10.17, 4.17, 2.83, and 2.67 min, respectively. The duration of the temperature increase decreased rapidly as the concentration of Fe^3+^ added increased, suggesting that the effects of the added concentration of ferric ions on the reaction heat release rate are extremely significant. The higher the concentration of ferric ions, the faster the reaction heat release rate and the shorter the time taken for the temperature to increase.

Fig. 5 shows that when the concentration of Fe^3+^ added increased from 5 mmol L^−1^ to 10 mmol L^−1^, *η* increased from 89.5% to 93.45%. Subsequently, when the concentration of Fe^3+^ added increased to 30 mmol L^−1^, *η* decreased to 82.67%. This showed that *η* first increased before decreasing, when the concentration of Fe^3+^ added increased.^21^ This was mainly due to the fact that when Fe^3+^ concentration was low, the reaction rate was slow; this inhibited the synthesis of ·OH and 
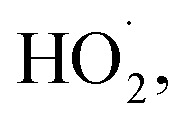
^22,23^ thereby affecting the reaction between the free radicals and *o*-phenylenediamine. As the concentration of Fe^3+^ added increased, the reaction rate and the number of free radicals produced per unit of H_2_O_2_ added increased, causing a subsequent rise in *η*. When the concentration of Fe^3+^ added was too high, the reaction rate of the processes in eqn (4)–(6), which are favorable to organic matter degradation, and the ones in eqn (7)–(9) that cause auto-consumption, increased simultaneously. This resulted in a rapid decrease in the reaction heat release duration *T*. When the auto-consumption reaction rate exceeds the organic matter degradation reaction rate, the degradation rate decreases.^18^

### 2.3 Effects of *o*-phenylenediamine concentration

**Fig. 6:**
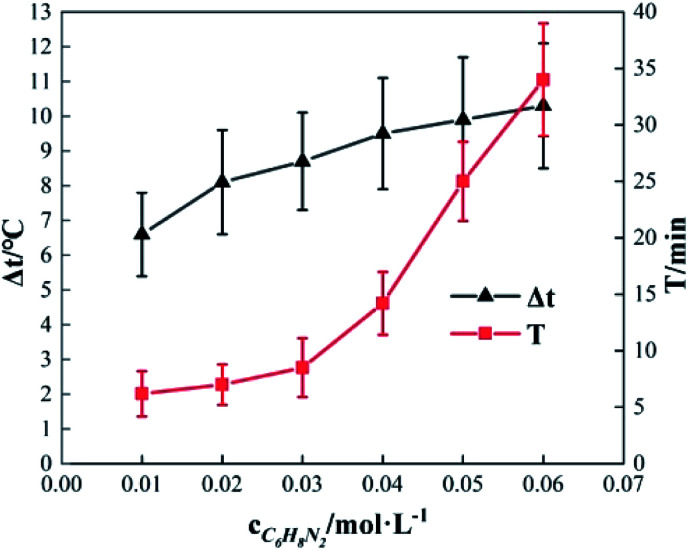
Effects of [C_6_H_8_N_2_] on Δ*t* and *T*.

**Fig. 7:**
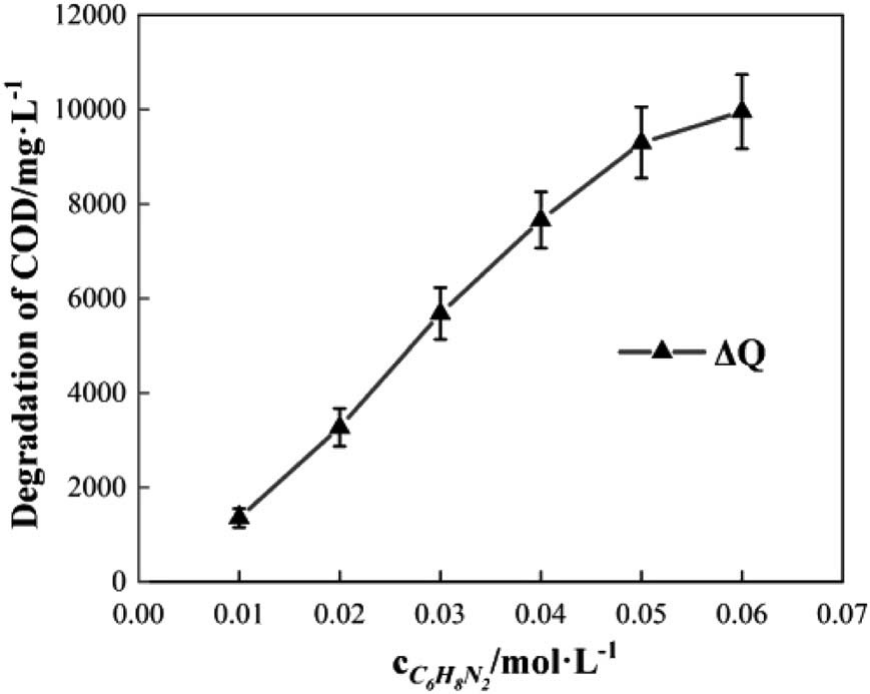
Effects of [C_6_H_8_N_2_] on Δ*Q*.

To study the effect of the initial concentration of *o*-phenylenediamine on Δ*t*, *T* and *η*, the reaction conditions were as follows: the volume of *o*-phenylenediamine solution was 200 mL, initial reaction temperature was 30 °C, pH was 7.8, concentration of H_2_O_2_ was 0.2 mol L^−1^, concentration of Fe^3+^ was 10 mmol L^−1^, and reaction time was 40 min. Fig. 6 and 7 depict the Δ*t*/*T* curves and *η* curves of the *o*-phenylenediamine solution with the initial concentration of *o*-phenylenediamine respectively. Fig. 6 and 7 show the Δ*t*/*T* and *η* curves of how *o*-phenylenediamine concentrations changed from initial levels when the initial reaction temperature was 30 °C, concentration of H_2_O_2_ added was 0.2 mol L^−1^, concentration of Fe^3+^ added was 10 mmol L^−1^, and duration of the reaction was 40 min. As the COD values for *o*-phenylenediamine solutions with different concentrations were different, Δ*Q* was used instead of *η* to more accurately reflect the degradation of these *o*-phenylenediamine solutions in the H_2_O_2_/Fe^3+^ system. Fig. 6 and 7, respectively, show that when [C_6_H_8_N_2_] is 0.01 mol L^−1^, Δ*t* is 6.6 °C, *T* is 6.2 min, and Δ*Q* is 1352.73 mg L^−1^; and when [C_6_H_8_N_2_] is 0.06 mol L^−1^, Δ*t* is 10.3 °C, *T* is 34 min, and Δ*Q* is 9956.62 mg L^−1^, the values of Δ*t*, *T*, and Δ*Q* increase as the initial concentration of *o*-phenylenediamine increases. This is because when the initial concentration of *o*-phenylenediamine is increased, there is an increased probability of collisions between *o*-phenylenediamine molecules and ·OH and 
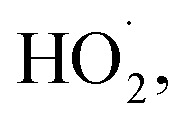
 and the number of molecules participating in the reaction and the heat released by the reaction increases, causing Δ*t* and Δ*Q* to increase. When the number of reactions between *o*-phenylenediamine and ·OH and 
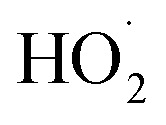
 increases, phase changes alleviate the occurrence of side reactions by the free radicals and increase the effective utilization rate of ·OH and 
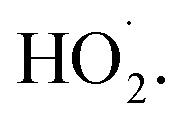
^24^

The rate of at which ·OH and 
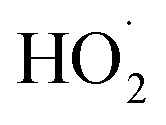
 are generated in the solution limits the reaction rate between the free radicals and *o*-phenylenediamine molecules. When the initial concentration of H_2_O_2_ and Fe^3+^ added to the solution are fixed, the initial reaction rate between ·OH and 
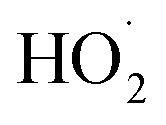
 and *o*-phenylenediamine is relatively stable. The greater the number of *o*-phenylenediamine molecules in the solution, the longer the time required for the reaction; this is evidenced as an increase in *T* as the initial concentration of *o*-phenylenediamine increases.

### 2.4 Effects of initial reaction temperature

**Fig. 8:**
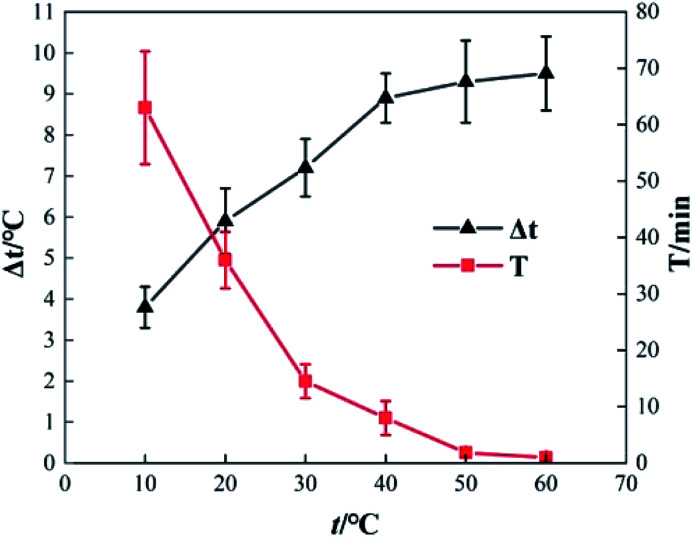
Effects of initial reaction temperature on Δ*t* and *T*.

**Fig. 9:**
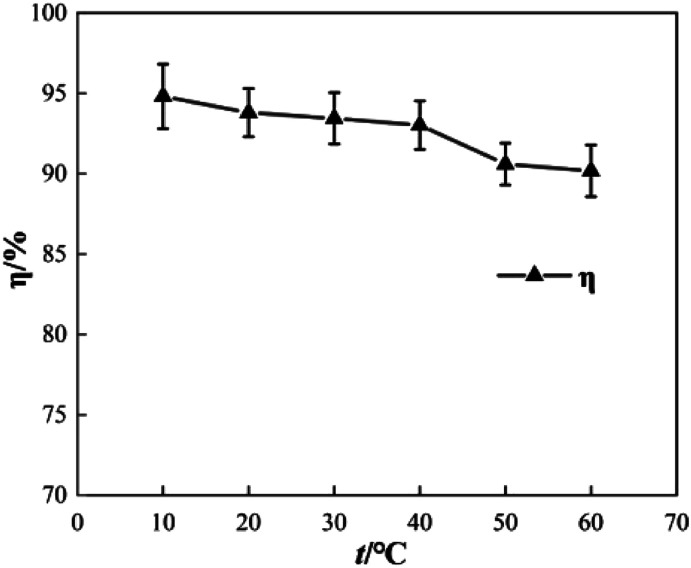
Effects of initial reaction temperature on *η*.

To determine the effect of initial reaction temperature on Δ*t*, *T* and *η*, the reaction conditions were as follows: the concentration of *o*-phenylenediamine solution was 0.04 mol L^−1^, volume was 200 mL, pH was 7.8, concentration of H_2_O_2_ was 0.2 mol L^−1^, Fe^3+^ was added at a concentration of 10 mmol L^−1^, and the reaction time was 60 min. Fig. 8 shows that Δ*t* increases as the initial reaction temperature increases and that *T* decreases rapidly as the initial reaction temperature increases. When the initial reaction temperature is 10 °C, *T* is 63 min; when the initial reaction temperature is 60 °C, *T* is 1 min. This occurs mainly because the number of activated molecules in the solution increases when initial reaction temperature increases, thereby accelerating the reaction rate. Fig. 9 shows that when *t* = 10 °C, *η* is 94.81% and when *t* = 60 °C, *η* is 90.18%, the *η* curve shows a decreasing trend when initial reaction temperature increases. This is mainly because the rates of auto-consumption caused by the processes described in eqn (7) and (8) and thermal decomposition of H_2_O_2_ to form O_2_ and H_2_O increase simultaneously owing to rapid oxidization and degradation of *o*-phenylenediamine by ·OH and 
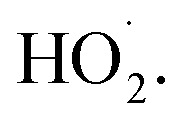
 This decreases the degradation rate of *o*-phenylenediamine).^24^

## 3. Discussion

### 3.1 Blank test analysis

**Fig. 10:**
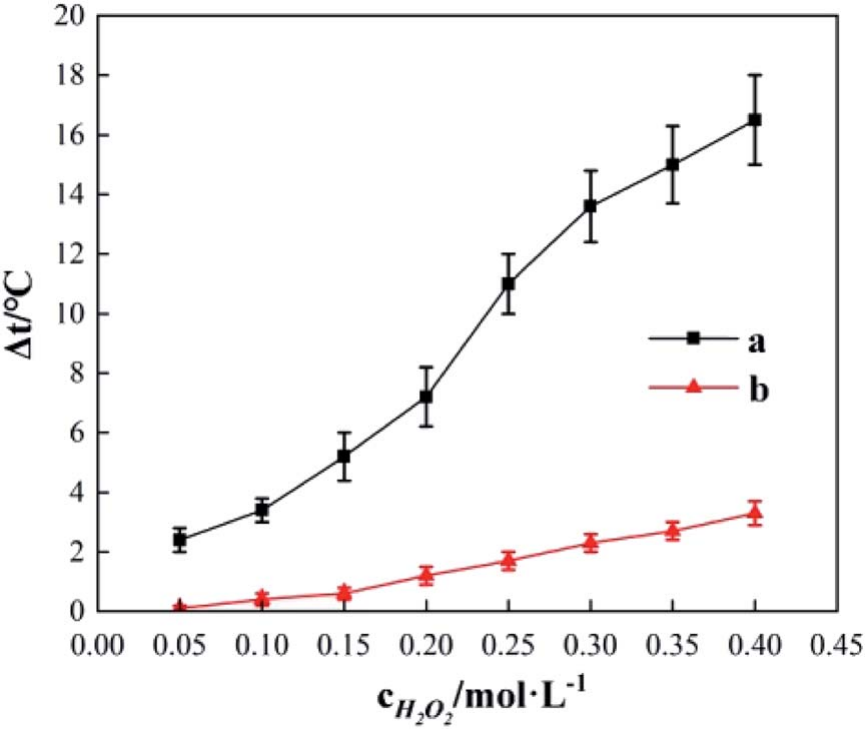
Comparison of temperature elevation between *o*-phenylenediamine solution and water.

Fig. 10 shows the Δ*t* curve when H_2_O_2_ and Fe_2_(SO_4_)_3_ are added to 200 mL of 0.04 mol L^−1^*o*-phenylenediamine and 200 mL of deionized water when the concentration of H_2_O_2_ added is 0.2 mol L^−1^, the concentration of Fe^3+^ added is 10 mmol L^−1^, and reaction duration is 30 min. Fig. 10 shows that temperature increased after H_2_O_2_ and Fe^3+^ were added to the *o*-phenylenediamine solution and deionized water. When [H_2_O_2_] is 0.4 mol L^−1^, the Δ*t* values of *o*-phenylenediamine solution and deionized water are 16.5 °C and 3.3 °C, respectively, and the magnitude of the increase in temperature of in the Fe^3+^/H_2_O_2_ quasi-Fenton system is significantly higher when *o*-phenylenediamine solution is used, compared to when deionized water is used. This shows that exothermic reactions occur when the Fe^3+^/H_2_O_2_ quasi-Fenton system degrades *o*-phenylenediamine and during auto-decomposition in deionized water. The amount of heat released by *o*-phenylenediamine degradation by ·OH and 
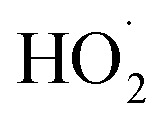
 is far greater than that by the auto-decomposition of these free radicals. The amount of heat released and the relationship between the reactions of production of ·OH and 
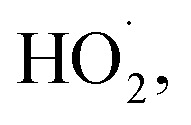
 their reaction with *o*-phenylenediamine, and auto-consumption require further study.

### 3.2 Environmental heat analysis

**Fig. 11:**
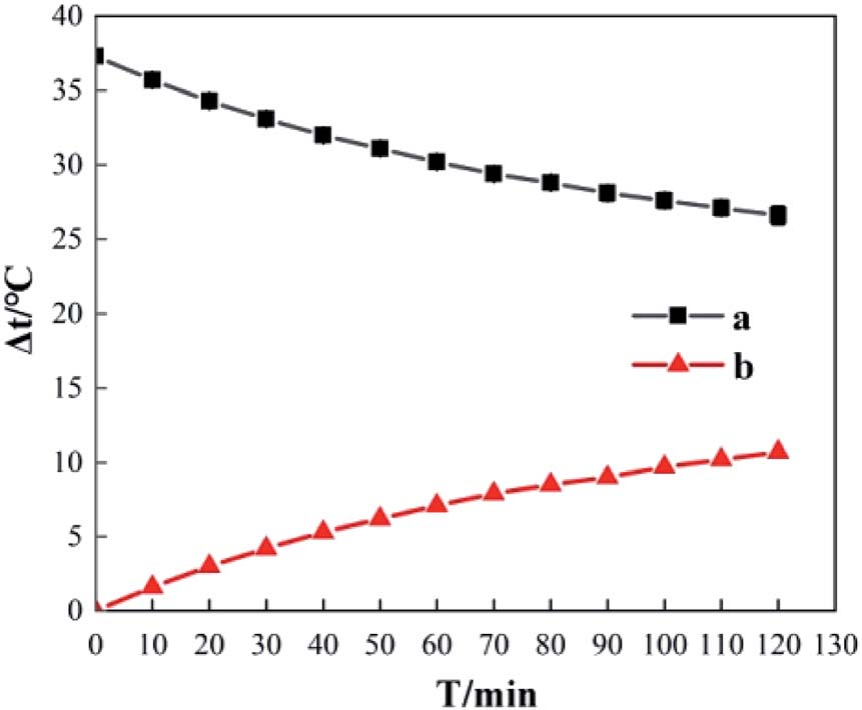
Environmental heat dissipation curve.

In Fig. 11, curve a shows the variation in temperature when the room temperature is 20.5 °C and the initial temperature of 200 mL deionized water is 37.3 °C in the experimental setup; curve b is the corresponding temperature reduction curve. Curve a shows that even though thermal insulation measures were adopted as much as possible in the experimental setup given in Fig. 1, the problem of heat dissipation to the surrounding environment still exists. In the experimental setup, the temperature of the deionized water gradually decreases as time increases; it reached 26.6 °C from the initial temperature of 37.3 °C in 120 min, a temperature reduction of 10.7 °C. The curve shows that the differences in temperature reduction gradually increase as the incubation time increases; the differences in temperature reduction for 10, 20, 30, and 120 min were 1.6 °C, 3 °C, 4.2 °C, and 10.7 °C, respectively. This shows that heat dissipation from the system to the surrounding environment is an important factor affecting temperature elevation data. When reaction time is longer, revisions in heat dissipation values are required for the temperature elevation data.

### 3.3 Temperature rise process analysis

**Fig. 12:**
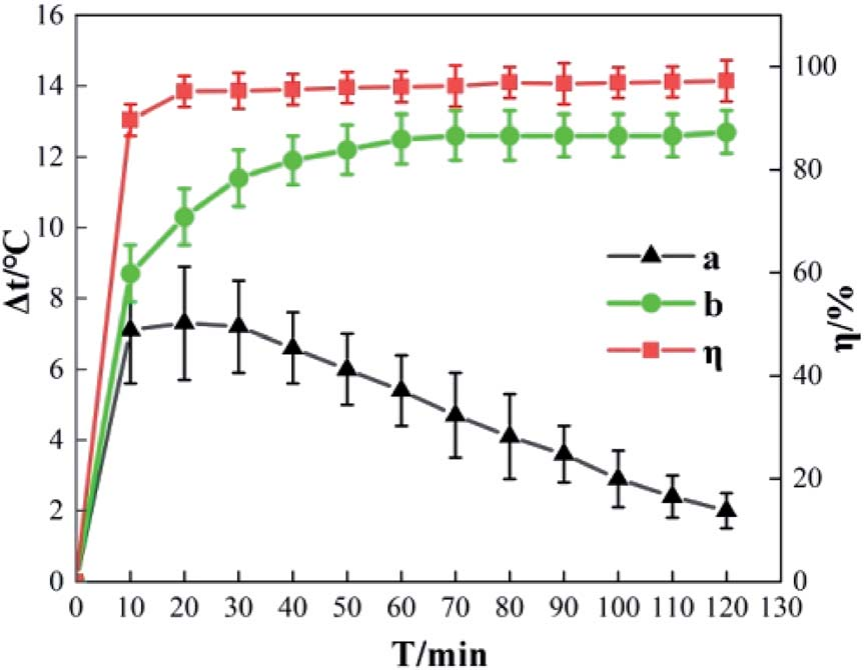
Effects of reaction time on Δ*t* and *η*.

Fig. 12 shows the Δ*t* and *η* curve in which *o*-phenylenediamine concentration changes with time when the concentration of H_2_O_2_ added is 0.2 mol L^−1^, the concentration of Fe^3+^ added is 10 mmol L^−1^, and the reaction duration is 120 min. Curve b is the measured Δ*t* curve and curve a is the Δ*t* curve after compensating for heat dissipation from the system to the surrounding environment. Curve a shows that during *o*-phenylenediamine degradation in the Fe^3+^/H_2_O_2_ quasi-Fenton system, the process by which the temperature of the solution increases can be divided into rapid, stable, and lag phases of temperature increase. In the first 10 min after the start of the reaction, Δ*t* and *η* increase rapidly as time progresses. When *T* is 10 min, Δ*t* is 8.7 °C and *η* is 89.62%. This is because during the early stages of the reaction the concentrations of *o*-phenylenediamine, H_2_O_2_, and Fe^3+^ in the solution are relatively high; therefore, the cyclic reaction between Fe^3+^, Fe^2+^, and H_2_O_2_ is faster and the ·OH and HO_2_ produced reacts with *o*-phenylenediamine. During *o*-phenylenediamine degradation, large amounts of heat are released simultaneously, causing Δ*t* and *η* to increase rapidly. This is the rapid phase of the temperature increase. As the reaction progresses, the concentration of *o*-phenylenediamine and H_2_O_2_ in the solution decreases rapidly, the reaction becomes weaker, and *t* and *η* show a slight increasing trend. When *T* is 20 min, the increases in *t* and *η* reach a critical point, curve a shows a decline in increasing trend, an inflection point appears on curve b and starts to decrease while *η* approaches its maximum. This shows that after 20 min of reaction, the speed of the heat release from the reaction system starts decreasing below the rate of heat dissipation to the surrounding environment. This is the stable phase of the temperature increase. Following that, the H_2_O_2_ in the solution is almost depleted as the reaction time increases; the reaction becomes extremely weak and the heat released by the system itself approaches 0. This stage is the lag phase of the temperature increase.

### 3.4 pH change analysis

**Fig. 13:**
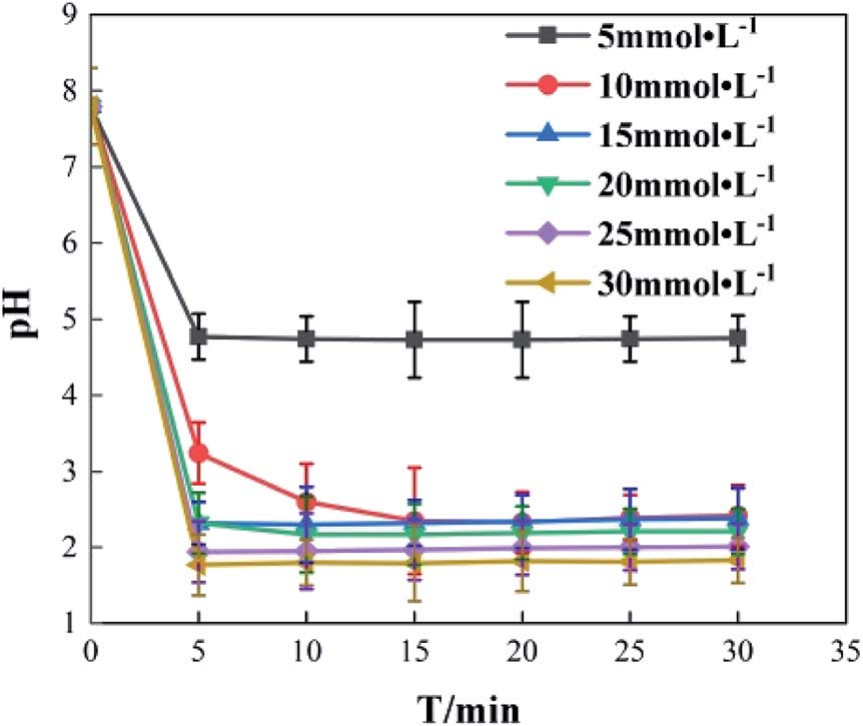
pH change.

It was found that Fe^3+^ is the main factor affecting the pH value of *o*-phenylenediamine solution, while H_2_O_2_ has little effect. Change of pH in the reaction process was studied under the following reaction conditions: concentration of *o*-phenylenediamine solution = 0.04 mol L^−1^, initial reaction temperature = 30 °C, pH = 7.8, concentration of H_2_O_2_ = 0.2 mol L ^−1^. pH change of an *o*-phenylenediamine solution after the addition of H_2_O_2_ and Fe^3+^ is shown in Fig. 13. pH of the *o*-phenylenediamine solution rapidly decreased after the addition of H_2_O_2_ and Fe^3+^. When Fe^3+^ was 5 mmol L^−1^, 10 mmol L^−1^ and 20 mmol L^−1^, the pH of the *o*-phenylenediamine solution was 4.8, 3.2 and 1.8, respectively; this can be attributed to the hydrolysis reaction of Fe^3+^ in the solution. As shown in eqn (10), the higher the Fe^3+^ concentration, the more H^+^ produced in the solution, resulting in lower pH of the solution.

10 Fe^3+^ + 3H_2_O_2_ → Fe(OH)_3_ + 3H^+^

The above results show that under conditions in which the concentrations of *o*-phenylenediamine and Fe^3+^ added are identical, Δ*t* rapidly increases as the concentration of H_2_O_2_ added increases. This shows that the concentration of H_2_O_2_ added is a main factor affecting the heat released by the Fe^3+^/H_2_O_2_ quasi-Fenton system. Moreover, under the same conditions, Δ*t* rapidly increases as the concentration of Fe^3+^ added increases. This shows that the concentration of Fe^3+^ added or the initial reaction temperature of the solution are the main factors affecting the heat released by the Fe^3+^/H_2_O_2_ quasi-Fenton system. The effects of the concentration of Fe^3+^ added on the exothermic reaction rate is mainly due to the acceleration of the cyclic reaction between Fe^3+^, Fe^2+^, and H_2_O_2_ that occurs when the concentration of Fe^3+^ added increases^25,26^ and more ·OH and 
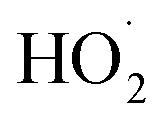
 are produced in the solution. This accelerates the degradation of organic matter. The initial reaction temperature affects reaction rate is because the number of activated molecules in the solution increases as temperature increases.

## 4. Conclusions and outlook

The study of the exothermic laws during H_2_O_2_/Fe^3+^ homogeneous Fenton degradation of *o*-phenylenediamine wastewater allowed us to draw the following conclusions:

(1) *o*-Phenylenediamine degradation by the H_2_O_2_/Fe^3+^ homogeneous Fenton system is accompanied by an increase in the temperature of the solution, and the concentration of H_2_O_2_ added is the main factor affecting solution temperature elevation. The increase in temperature of the solution occurs as the concentration of H_2_O_2_ increases. However, the concentration of Fe^3+^ added and the initial reaction temperature of the solution are the main factors affecting the exothermic reaction rate of the Fe^3+^/H_2_O_2_ quasi-Fenton system. The greater the concentration of Fe^3+^ added to the system, or the higher the initial reaction temperature, the faster the exothermic reaction rate. When [H_2_O_2_] is 0.2 mol L^−1^, Fe^3+^ is 10 mmol L^−1^, initial reaction temperature is 30 °C, reaction duration is 30 min, Δ*t* is 7.2 °C, and *η* is 93.45% when the concentration of 200 mL *o*-phenylenediamine is 0.04 mol L^−1^.

(2) The heat produced during the degradation of *o*-phenylenediamine by the Fe^3+^/H_2_O_2_ quasi-Fenton system originates mainly from the exothermic reaction between the ·OH and 
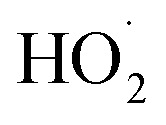
 and *o*-phenylenediamine molecules, its intermediate products, and the exothermic reaction when active groups undergo auto-consumption.

This paper summarizes the exothermic laws applicable when wastewater containing *o*-phenylenediamine is degraded by a Fe^3+^/H_2_O_2_ homogeneous quasi-Fenton system, and has positive significance for reducing wastewater treatment costs and improving energy utilization. However, there is still insufficient research on the amount and proportion of heat released in the various stages. Future studies will examine the heat released during these stages from the mechanism by which *o*-phenylenediamine is degraded by ·OH and 
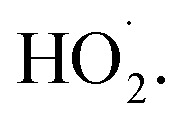


## Conflicts of interest

There are no conflicts to declare.

## Supplementary Material
